# Evaluating the effectiveness of the smartphone app, Drink Less, compared with the NHS alcohol advice webpage, for the reduction of alcohol consumption among hazardous and harmful adult drinkers in the UK at 6‐month follow‐up: protocol for a randomised controlled trial

**DOI:** 10.1111/add.15287

**Published:** 2020-11-26

**Authors:** Claire Garnett, Melissa Oldham, Colin Angus, Emma Beard, Robyn Burton, Matt Field, Felix Greaves, Matthew Hickman, Eileen Kaner, Gemma Loebenberg, Susan Michie, Marcus Munafò, Elena Pizzo, Jamie Brown

**Affiliations:** ^1^ Department of Behavioural Science and Health University College London London; ^2^ School of Health and Related Research University of Sheffield Sheffield; ^3^ Public Health England London; ^4^ Department of Psychology University of Sheffield Sheffield; ^5^ Department of Primary Care and Public Health Imperial College London London; ^6^ Bristol Population Health Science Institute University of Bristol Bristol; ^7^ Population Health Sciences Institute Newcastle University Newcastle upon Tyne; ^8^ Department of Clinical, Educational and Health Psychology University College London London; ^9^ School of Psychological Science University of Bristol Bristol; ^10^ Department of Applied Health Research University College London London

**Keywords:** Alcohol reduction, behaviour change, digital, intervention, protocol, RCT, smartphone app, UK

## Abstract

**Background and Aims:**

Digital interventions are effective for reducing alcohol consumption but evidence is limited regarding smartphone apps. Drink Less is a theory‐ and evidence‐informed app to help people reduce their alcohol consumption that has been refined in terms of its content and design for usability across the sociodemographic spectrum. We aim to evaluate the effectiveness and cost‐effectiveness of recommending Drink Less at reducing alcohol consumption compared with usual digital care.

**Design:**

Two‐arm individually randomised controlled trial.

**Setting:**

Online trial in the United Kingdom (UK).

**Participants:**

Hazardous or harmful drinkers (Alcohol Use Disorders Identification Test [AUDIT] score ≥8) aged 18+ who want to drink less alcohol (*n* = 5562). Participants will be recruited from July 2020 to May 2022 using multiple strategies with a focus on remote digital methods.

**Intervention and comparator:**

Participants will be randomised to receive either an email recommending that they use Drink Less (intervention) or view the National Health Service (NHS) webpage on alcohol advice (comparator).

**Measurements:**

The primary outcome is change in self‐reported weekly alcohol consumption, assessed using the extended AUDIT‐Consumption, between baseline and 6‐month follow‐up. Secondary outcomes include change in self‐reported weekly alcohol consumption assessed at 1‐ and 3‐month follow‐ups, and the proportion of hazardous drinkers; alcohol‐related problems and injury; health‐related quality of life; and use of health services assessed at 6‐month follow‐up. Effectiveness will be examined with adjusted regression models, adjusting for baseline alcohol consumption and using an intention‐to‐treat approach. A mixed‐methods process evaluation will assess engagement, acceptability and mechanism of action. Economic evaluations will be conducted using both a short‐ and longer‐term time horizon.

**Comments:**

This study will establish the effectiveness and cost‐effectiveness of the Drink Less app at reducing alcohol consumption among hazardous and harmful adult drinkers and will be the first randomised controlled trial of an alcohol reduction app for the general population in the United Kingdom. This study will inform the decision on whether it is worth investing resources in large‐scale implementation.

## INTRODUCTION

Hazardous and harmful alcohol consumption is a major public health concern and contributes to health inequalities with the most deprived groups suffering the most harm from alcohol [1]. Fewer than 7% of hazardous and harmful drinkers receive face‐to‐face interventions in primary care to support alcohol reduction [2] with key barriers to the delivery of these interventions by practitioners being lack of time and low confidence about discussing alcohol with patients [3, 4]. Digital interventions, such as websites and smartphone apps, may be effective for reducing alcohol consumption [5] and may overcome barriers to delivery of face‐to‐face interventions because they potentially have a broad reach and relatively low implementation costs (once developed) and therefore can be delivered at scale [6]. As digital technologies become more integrated into everyday life and widely used, digital interventions can have a large positive impact on public health and well‐being at a population level. Smartphone apps are a promising mode of intervention delivery because smartphones have become increasingly affordable to end users and prevalent among the United Kingdom (UK) population [7]. However, most digital alcohol interventions that have been evaluated are web‐based and there is little evidence on the effectiveness of apps. The few trials of apps have been based in other countries and usually with younger adults [8, 9, 10, 11]. Therefore, there is an urgent need for a robust evaluation of an evidence‐ and theory‐informed alcohol reduction app, which, if effective, could be widely recommended to drinkers in the UK. The Drink Less app was designed to help people reduce their alcohol consumption and has been developed and refined using a systematic and iterative process [12, 13]. The app is ready for a definitive evaluation to establish whether recommending it to people is more effective than usual digital care for the reduction of alcohol consumption.

Digital interventions—primarily web‐based—may reduce alcohol consumption, with an average reduction of 23g of alcohol (2.9 UK units) per week compared with participants in the control group [5]. In this Cochrane review of 42 randomised controlled trials (RCTs), only one of the digital interventions used a smartphone app, and this RCT was conducted in Sweden among university students, which found that the app may have led to a negative effect among men [8]. Updates of this review found a further three studies that used smartphone apps: one in Sweden among university students (that found the app had potential for reducing excessive alcohol use) [9], one for the general population in Canada (that found that access to the app had no impact on drinking) [10] and another for young adults in Australia (that found a decrease in the typical number of drinks on a drinking occasion in both conditions) [11]. Therefore, despite the availability of hundreds of alcohol‐related apps, none have been evaluated in a RCT among the general population of adults in the United Kingdom. The majority have also been developed without reference to scientific evidence or theory [14]. The lack of evidence highlights the necessity of a robust and pragmatic evaluation of an evidence‐ and theory‐informed alcohol reduction app, which, if effective, could be widely recommended.

Drink Less is an evidence‐ and theory‐informed app that aims to help hazardous and harmful drinkers reduce their alcohol consumption. Drink Less is one of the most popular alcohol reduction apps on the UK Apple app store and is highly rated by users, and is capable of reaching a large proportion of the UK population at a low incremental cost. The development and evaluation of Drink Less was guided by the Medical Research Council's guidance on complex interventions [15] and the Multiphase Optimisation Strategy [16]. The development of the Drink Less app was informed by the COM‐B model of behaviour [17] and multiple sources of evidence [14, 18, 19] meaning it has a robust theoretical basis and evidence base. The COM‐B model posits that behaviour results from interactions between ‘capability’, ‘opportunity’, and ‘motivation’, and was used to provide an overarching theoretical structure [17]. The empirical evidence and theoretical framework provided the basis for the prioritisation of intervention components to include in the app. Drink Less consists of seven key modules: (i) Goal Setting; (ii) Self‐Monitoring and Feedback; (iii) Normative Feedback; (iv) Cognitive Bias Retraining; (v) Action Planning; (vi) Behavioural Substitution; and (vii) Information About Antecedents. Details of these modules are reported in full elsewhere [12]. A factorial screening trial was conducted to identify the most effective modules (distinct behaviour change interventions) within the app, which established that four of the five original modules appeared to have an effect on reducing alcohol consumption after 4 weeks if combined with one of the other modules [13]. Data also suggested that users of Drink Less found it to be engaging [13], which is important to reduce participant attrition. The strategy for refining the effectiveness and usability of the app was based on: (i) findings from the previous factorial trial; (ii) a content analysis of user feedback (that indicated that users found it helpful in reducing their drinking); and (iii) an updated evidence review and meta‐analysis of behaviour change techniques in digital alcohol interventions. The refinement process is reported in full elsewhere (under review). Both the initial development and refinement of Drink Less have involved input from users across the social spectrum on the functionality, design and language used in the app [20]. The same approach was used for a smoking cessation digital intervention that was found to be effective for increasing smoking cessation rates across different socioeconomic groups [21].

The next step in the Multiphase Optimisation Strategy is to conduct a RCT to evaluate the long‐term effectiveness and cost‐effectiveness of the digital recommendation of the refined Drink Less app, compared with alcohol advice from the National Health Service (NHS) alcohol advice webpage (usual digital care, available to anyone seeking alcohol support), in reducing alcohol consumption among hazardous and harmful drinkers. This research will be the first RCT of an alcohol reduction app for the general population in the United Kingdom and will evaluate whether it is worth investing resources into promoting and disseminating the app on a larger scale. The aim of this trial is to evaluate the effectiveness and cost‐effectiveness of recommending Drink Less at reducing alcohol consumption compared with usual digital care.

### Research questions

1. Among hazardous and harmful drinkers, does the digital recommendation to use Drink Less compared with the NHS alcohol advice webpage reduce weekly alcohol consumption (in UK standard units) between baseline and 6‐month follow‐up?
2. Among hazardous and harmful drinkers, does the digital recommendation to use Drink Less compared with the NHS alcohol advice webpage:
  - Reduce weekly alcohol consumption (in UK standard units) between baseline and 1‐ and 3‐month follow‐up?
  - Reduce heavy episodic alcohol consumption at 6‐month follow‐up?
  - Reduce the proportion of hazardous drinkers at 6‐month follow‐up?
  - Reduce alcohol‐related problems and injury, and use of healthcare services at 6‐month follow‐up?
  - Improve health‐related quality of life at 6‐month follow‐up?
3. What is the extent of user engagement with Drink Less and does user engagement moderate these outcomes?
4. Through what psychological measures does engagement with Drink Less change drinking behaviour?
5. What are participants' views on the acceptability of the intervention?
6. What is the cost‐utility and potential impact on health inequalities of Drink Less compared with the NHS alcohol advice webpage in terms of reduction in alcohol consumption and health‐related quality of life using a short time horizon?
7. What is the longer‐term cost‐effectiveness and potential impact on health inequalities of Drink Less compared with the NHS alcohol advice webpage, if rolled out on a national level through active promotion to the public, over a 20‐year period?

## METHODS

### Design

A two‐arm, parallel group, RCT with a 1:1 allocation comparing the recommendation to use the intervention (Drink Less) with usual digital care (the NHS alcohol advice webpage), with an embedded mixed‐methods process evaluation.

### Setting

The study will take place online with participants who live in the UK.

### Participants

Participants will be included if they: are aged 18 years or over, live in the UK, are hazardous and harmful drinkers (Alcohol Use Disorders Identification Test (AUDIT) score ≥8), have access to an iOS device (i.e. iPhone, iPod touch or iPad, because Drink Less is currently only available on these devices) and want to drink less alcohol. Participants will be excluded if they are unwilling to complete follow‐up assessments or are unable to read English (for pragmatic reasons), which will be assessed with a Yes/No question in the screening questionnaire.

### Recruitment

Recruitment is due to run from July 2020 to March 2022 via a multi‐pronged strategy including: an advertisement on the NHS website; a mail‐out to a database of UK‐based users of the Smoke Free app; and press releases and local advertising through health care providers and/or national and local government colleagues. The advertisements will be co‐developed with public representatives.

### Sample size

A sample size of 5562 participants (2781 in the comparator group and 2781 in the intervention group) is required to detect a mean difference reduction of 2 UK units (16 g of alcohol) in weekly alcohol consumption (the primary analysis) at 90% power with an α of 0.05 and a two‐tailed test. This was calculated using G*Power software [22]. The estimated effect size is in line with the Cochrane review on digital alcohol interventions [5] and is roughly equivalent to that found in face‐to‐face brief interventions [23].

An initial sub‐sample of 26 participants (13 from each group [24]), who consented to a short interview about their experience of the trial, will be selected after the 6‐month follow‐up, as part of the mixed‐methods process evaluation. Participants will be purposively sampled to achieve good diversity in terms of sociodemographic characteristics and with high and low engagement. Data will be analysed and data collection will continue only if thematic ‘meaning’ saturation is not reached (see Analysis). Collection will continue in an iterative process (adding 10 participants at a time) until saturation is reached.

### Intervention

**Figure 1:**
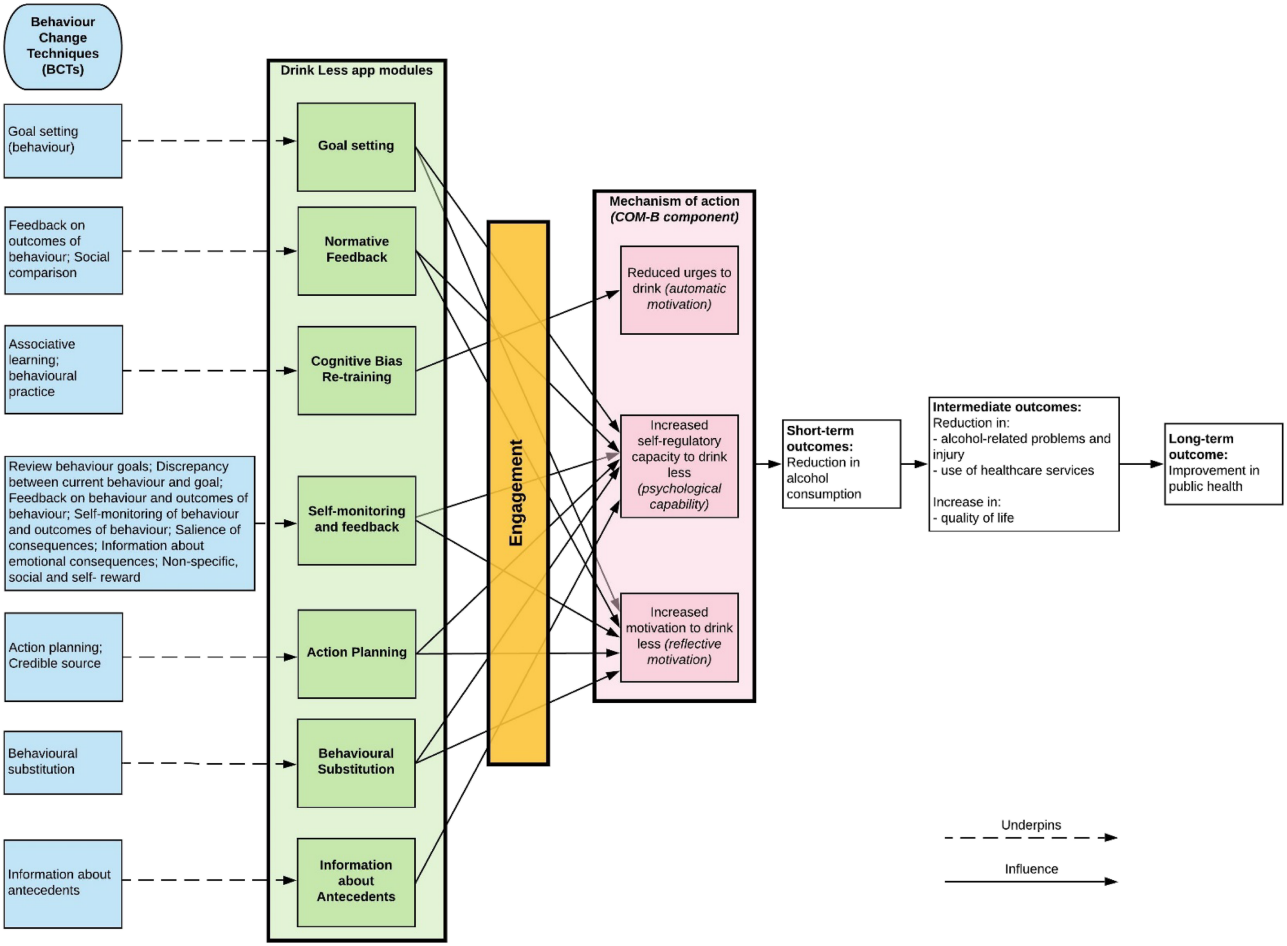
Logic model for the process of change of the Drink Less app (developed by the internal research team). [Colour figure can be viewed at wileyonlinelibrary.com]

Drink Less is a stand‐alone app‐based intervention that is freely available via the Apple app store in the UK [25]. Drink Less was developed for hazardous and harmful drinkers to help them reduce their alcohol consumption. Drink Less consists of evidence‐based modules to help users change their drinking behaviour: Goal Setting, setting weekly ‘drinking reduction’ goals; Self‐Monitoring and Feedback, monitoring alcohol consumptions and seeing progress on goals; Action Planning, creating plans for dealing with difficult drinking situations; Normative Feedback, providing personalised feedback on how an individual's drinking behaviour compares to the norm; Cognitive Bias Re‐Training, a game for retraining users' automatic biases for alcoholic drinks; Behavioural Substitution, planning to substitute drinking with a neutral behaviour; and Information about Antecedents, providing users with information about situations and events, emotions and cognitions that predict their drinking. These evidence‐based modules map to behaviour change techniques (Fig. 1). The development and content of the original Drink Less version is reported in full elsewhere [12] and the refined version is reported online (https://osf.io/mc8yz/). The app contains standard features such as the UK Chief Medical Officers' low‐risk drinking guidelines (14 units a week) [26].

On downloading the app, users are asked to complete the AUDIT, provide sociodemographic details and then receive the Normative Feedback module. Users are then guided through setting a goal and shown how to use the key features of the app. Users can access all of the modules from the dashboard and the menu bar. The dashboard (the landing page of the app) has suggestions for the user to complete each day, as well as features of and links to the modules. Users can choose to have daily reminders to complete their drinks and mood diary for the previous day. The app provides a ‘toolbox’ of features for users to choose from and access as and when they want. The app is not tailored to the user except for personalised feedback in two modules: Normative Feedback and Self‐Monitoring and Feedback. Any modifications to the app during the trial (e.g. bug fixes) will be documented and reported.

Drink Less is expected to reduce the alcohol consumption of its users based on (i) its robust theoretical basis (the COM‐B model of behaviour) [17]; (ii) its evidence base; and (iii) user feedback that indicates users believe that it helps them to reduce their drinking and has a positive effect on their health and well‐being. It is also expected to reduce urges to drink, increase motivation to drink less and increase self‐regulatory capacity to drink less (see Fig. 1 for the logic model). Drink Less is also highly rated by users (average 4.2‐star rating in the Apple (UK) App Store with over 60 000 unique users as of 1 November 2019).

### Comparator

The comparator group will receive the recommendation to view the NHS alcohol advice webpage on ‘Tips on cutting down’ that also has links to other webpages aimed at hazardous and harmful drinkers [27]. This can be considered reflective of ‘usual digital care’ in this context because it is the digital support currently available to treatment‐seeking individuals from the NHS. Therefore, this comparator best serves the primary purpose of the trial [28], which is to investigate whether it is worth promoting Drink Less over the ‘usual digital care’ and is of direct policy relevance. Furthermore, it is important to have a comparator that is relevant to the same target population as the intervention, and both Drink Less and the NHS webpage are aimed at adults in the general population. Any changes to the comparator during the trial will be documented.

This is a pragmatic trial where participants have access to a wide range of digital support and therefore it is not possible to simply compare use of the Drink Less app with use of the NHS webpage to assess which is more effective. Instead, the pragmatic nature of the trial means the evaluation is in terms of whether the recommendation of the Drink Less app enhances usual digital care over and above what is currently used by the participant.

### Procedure

**Figure 2:**
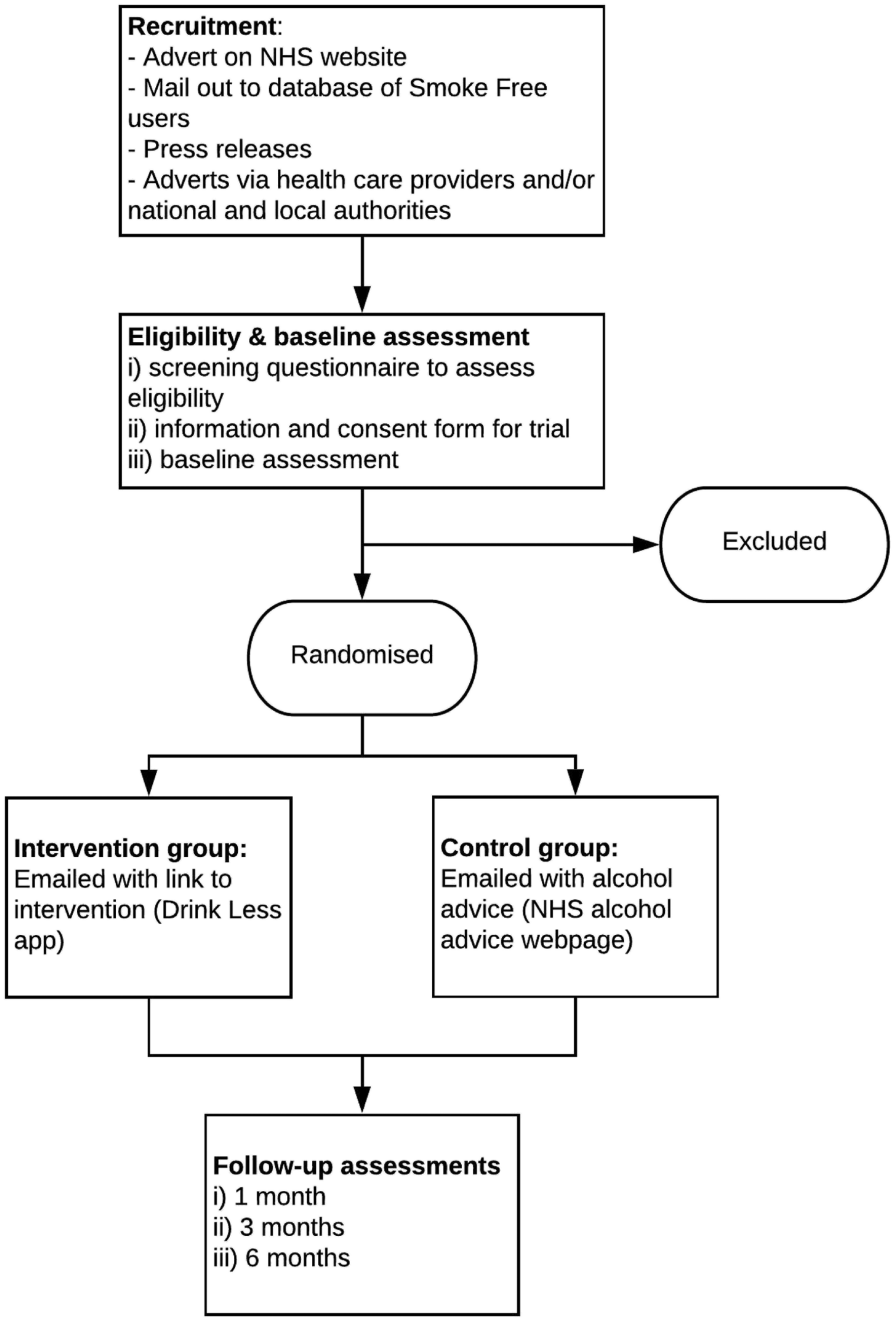
Flowchart of procedure

Figure 2 illustrates the study design and flow of participants and Table 1 summarises the schedule of enrolment and follow‐up assessment for trial participants.

**Table 1:** Schedule of enrolment and follow‐up assessments

Assessment	Time‐point			
	Baseline	1 month	3 months	6 months
Informed consent	x			
Eligibility screening	x			
Randomisation	x			
Intervention/comparator initiation	x			
Sociodemographic characteristics^a^	x			
Weekly alcohol consumption	x	x	x	x
AUDIT score and proportion of hazardous drinkers	x			x
Alcohol‐related problems or consequences and alcohol‐related injury				x
Use of healthcare services				x
Health‐related quality of life				x
Psychological measures	x			x
Engagement	x	x	x	x
Acceptability				x
Adverse events		x	x	x
Debriefing				x

^a^ Sociodemographic characteristics include: age, sex, ethnicity, education, occupation, income.

#### Eligibility assessment

Participants will self‐enrol into the study and potential participants will be asked to respond to a web‐based screening questionnaire to assess the inclusion and exclusion criteria, including the full AUDIT with the timeframe of the past 6 months (see Measures).

#### Consenting

If people meet the eligibility criteria they will be shown the information sheet (https://www.ucl.ac.uk/epidemiology‐health‐care/sites/epidemiology‐health‐care/files/participant_information_sheet.pdf) and informed that they will be re‐contacted on three occasions (at 1, 3 and 6 months). Participants will then be asked to provide consent online to participate in the study.

#### Baseline assessment

Participants will complete a web‐based assessment of sociodemographic measures (see Measures) and their contact details (email address, telephone number and postal address) for follow‐up assessments.

#### Randomisation

Participants who complete the baseline assessment will be randomised individually to intervention and comparator groups using block randomisation (block size of 50) and a random allocation sequence generated by an online automated algorithm (at a ratio of 1:1). Participants will be blinded to study arm. There will be no involvement of the researchers in the randomisation process, and there will be complete allocation concealment.

#### Intervention and comparator delivery

Participants will be emailed within 24 hours of the baseline assessment and randomisation with the recommendation to either use Drink Less (the intervention) or the NHS alcohol advice webpage (comparator). Participants allocated to the intervention condition will be provided with instructions on how to download the Drink Less app. Participants in both conditions will be provided with the contact details of the project team who will provide ongoing technical support, if required, during office hours. These emails will be co‐developed with public representatives.

#### Follow‐up assessments

Follow‐up assessments will be conducted 1, 3 and 6 months after baseline. The 6‐month follow‐up assessment will assess the primary and secondary outcome measures and psychological measures; the follow‐up assessments at 1 and 3 months will assess the self‐reported weekly alcohol consumption only. Participants will have up to 30 days to complete each survey to maximise data retention. Participants will be sent up to three automated emails with a link to a web‐based survey for the follow‐up assessments on days 0, 5 and 9. Participants who do not complete the web‐based follow‐up assessment will be sequentially offered opportunities to do so via phone (called once per day on days 10–17), mailed survey (on day 18) and by postcard (on day 30). Participants will be compensated with gift vouchers of up to £36 for completing the three surveys: £6 for the survey at 1 and 3 months; £12 at 6 months with an additional £12 if the 6‐month survey is completed within 24 hours.

At the 6‐month follow‐up, participants will indicate whether they are happy to be called for a short interview about their experience of the trial; there is no additional compensation for this telephone interview. Acceptability will then be measured after the 6‐month follow‐up via telephone interviews.

Participants will be asked whether they experienced any unexpected consequences, adverse events or other harms from participating in the study (in an open‐ended question at the 1‐, 3‐ and 6‐month follow‐up) and whether they have used any other forms of support for alcohol reduction (at the 6‐month follow‐up).

#### Debriefing

On completion of the trial, after the final follow‐up at 6 months, the comparator group will also be informed about the intervention, Drink Less.

### MEASURES

#### Sociodemographic measures

Sociodemographic measures will be assessed at baseline: age (in years, continuous), sex (% female), ethnicity (% white), education (% post‐16 educational qualifications), occupation (to derive social grade AB, C1, C2, D, E dichotomised into: ABC1 [managerial, professional and intermediate occupations] vs. C2DE [skilled, semi‐skilled, unskilled manual and lowest‐grade worked or unemployed]) and annual household income (% >£26 000 [29]).

### COVID‐19 measures

The recent COVID‐19 pandemic is affecting many aspects of people's lives in the UK. A lockdown is currently in place, limiting many people's ability to leave their homes, apart from essential journeys. Early evidence suggests that COVID‐19 may affect alcohol consumption, some lighter drinkers are drinking less than usual and some heavier drinkers are drinking more [30, 31, 32, 33]. To assess for the effects of the pandemic in the analysis, participants will respond to a brief COVID‐19 survey at each time point. Users will be asked ‘Do you currently feel like COVID‐19 is affecting your alcohol consumption and how you feel about drinking alcohol?’ If participants respond ‘no’ they will continue with the rest of the survey. Participants responding ‘yes’ will be asked to answer five follow‐up questions assessing the extent to which the pandemic is affecting their concerns about their alcohol consumption, their motivation to cut down and their patterns of consumption. Change in concerns about drinking will be measured by the question ‘Is COVID‐19 and its associated effects (e.g. financial, social or health) currently affecting how worried you feel about your alcohol consumption?’ followed by three response options: ‘more worried’, ‘no change’ and ‘less worried’. Change in motivation to reduce alcohol consumption will be measured by the question ‘Is COVID‐19 and its associated effects currently affecting your motivation to reduce your alcohol consumption?’ with three response options: ‘more motivated’, ‘no change’ and ‘less motivated’. Three questions measure changes in drinking patterns. Change in the frequency of drinking is measured by the question ‘Is COVID‐19 and its associated effects affecting how frequently you consume alcohol?’ with three response options: ‘consume alcohol more frequently’, ‘no change’ and ‘consume alcohol less frequently’. Change in the volume of alcohol consumed is measured by the question ‘Is COVID‐19 and its associated effects currently affecting how many units of alcohol you generally consume when you do drink?’ with three response options: ‘generally drink more units’, ‘no change’ and ‘generally drink less units’. Finally, change in the frequency of binge drinking is measured by the question ‘Is COVID‐19 and its associated effects currently affecting how often you consume 6 or more units of alcohol on a single occasion?’ with three response options: ‘more likely to consume 6 or more units on a single occasion’, ‘no change’ and ‘less likely to consume 6 or more units on a single occasion’. The date of national responses to COVID‐19 (e.g. lockdown) will also be monitored and recorded by the research team.

#### Primary outcome measure

The primary outcome measure is change between baseline and 6‐month follow‐up in self‐reported weekly alcohol consumption estimated over the last month, in UK standard units. Change in weekly alcohol consumption will be derived from the extended quantity‐frequency questions of the AUDIT [34], adjusting for heavy episodic use (question 3 of the AUDIT) and therefore allocating an individual to a category of consumption that is closest to their actual consumption. The AUDIT‐C questions exhibit similar sensitivity and specificity to the full AUDIT [35] and have demonstrated excellent reliability and responsiveness to short‐term change [36]. The extended AUDIT‐C is a variant of the AUDIT‐C, which has a greater range of response options (seven instead of five) on the quantity and frequency items, therefore improving the granularity of the information collected. This method of deriving alcohol consumption has been used in other trials [37, 38, 39, 40] and has high levels of agreement in levels of self‐reported consumption when compared with other retrospective daily diary measures [41]. This measure minimises response burden on participants because of its brevity, which is a critical issue in digital trials that have minimal contact with participants and can suffer from high levels of attrition [42].

#### Secondary outcome measures

- Change between baseline and 1‐ and 3‐month follow‐ups in self‐reported weekly alcohol consumption estimated over the last 1 and 2 months, respectively;
- Heavy episodic alcohol use (measured using AUDIT question 3) at 6‐month follow‐up
- Proportion of hazardous drinkers (AUDIT score ≥8);
- Alcohol‐related problems or consequences and alcohol‐related injury (measured using the Alcohol Short Index of Problems) [43] at 6‐month follow‐up;
- Use of healthcare services (measured using the Service Use Questionnaire) [44, 45] at 6‐month follow‐up;
- Health‐related quality of life (measured using the EQ‐5D‐5L) at 6‐month follow‐up.

The self‐report AUDIT questionnaire has 10 items that measure alcohol consumption, harms and dependence. The AUDIT is a reliable and standardised alcohol‐related outcome measure that is commonly used in alcohol trials [5, 23]. It has a high test–retest reliability when completed online [46], allows the derivation of a core outcome set for consistency across trials and minimises research waste and selective reporting [47]. The timeframe of the AUDIT will be modified to refer to the past 6 months when asked at the 6‐month follow‐up so this can be directly compared with the baseline AUDIT, which will also refer to the past 6 months. The extended AUDIT‐C when asked at the 1‐ and 3‐month follow‐up will refer to the past 1 month and 2 months, respectively, to capture participants’ alcohol consumption since the last survey.

#### Process measures

The mixed‐methods process evaluation involves assessing psychological measures, engagement and acceptability.

Psychological measures will be assessed as potential mechanisms of action at baseline and 6‐month follow‐up using four theoretical measures: urges to drink; motivation to drink less; self‐regulatory and self‐monitoring capacity (see Fig. 1 for the Logic Model). Strength of urges to drink will be measured by the question ‘How strongly have you felt the urge to drink alcohol in the past 24 hours?’ with six options from ‘Not at all’ to ‘Extremely strong’. The strength of urges to drink measure has been chosen because it can be done through a single item question and has fair long‐term test–retest reliability [48]. The motivation to drink less will be measured with the single‐item motivation to stop scale (MTSS). The MTSS and the urges to drink measure are both used in the Alcohol Toolkit Study allowing for national comparisons and have been successfully used in an observational study that estimates patterns of alcohol consumption and reduction in a sample in England [49]. Self‐regulatory capacity will be measured by ‘How difficult do you find it to control your drinking?’ using a 5‐point scale from ‘Not at all’ to ‘Extremely’. Self‐monitoring capacity will be measured by ‘How often, if at all, do you keep track of how many units of alcohol you personally drink each week?’ ranging from ‘Never’ to ‘Always’.

Engagement with Drink Less will be assessed in terms of app download, frequency, amount, duration and depth of engagement [50]—all automatically recorded within the app, among participants in the intervention group. This will provide objective data on how participants interact with the app. App download will be assessed by whether the participant downloaded and opened Drink Less (a binary yes/no measure). Frequency of engagement will be assessed by number of sessions, where a new session is defined as a new screen view after 30 minutes of inactivity [51]. Amount of engagement will be assessed by time on app in minutes. Duration of engagement will be assessed by number of days used. Depth of engagement will be assessed by the percentage of available screens viewed. Participants will also be asked about their use of the app in the semi‐structured interviews to complement and enhance the patterns identified from the objective engagement data. For example: ‘Can you tell me about your experience of using the app?’ and ‘In what situations did you use it and why?’ Adherence to either the intervention or comparator will be measured at 1‐month follow‐up with the self‐report question ‘Did you look at or use the digital tool we recommended?’ with options Yes/No. Participants are informed that their answer itself does not matter and that they are still eligible to take part, but that their answer will help the researchers to draw more accurate conclusions.

The acceptability of the intervention will be assessed in a number of short semi‐structured interviews among a sub‐sample of participants in both the intervention and comparator group after the 6‐month follow‐up. The interview will focus on perceptions of the intervention in terms of the acceptability of the app—the extent to that participants consider the app to be appropriate, based on anticipated or experienced cognitive and emotional responses to the intervention [52]. The interview topic guide will be based on the Theoretical Framework of Acceptability [52].

#### Economic measures

Unit costs for the economic evaluation will be taken from standard sources (e.g. Personal Social Services Research Unit [PSSRU] and NHS tariffs).

### Data management and monitoring

Baseline and follow‐up assessment data will be collected online in Qualtrics and then held securely in Data Safe Haven. All personal data will be pseudonymised. Engagement data will be collected automatically from the app and downloaded via python/pandas script into Data Safe Haven from a secure https protocol and ‘Nodechef’ (an online platform for hosting mobile apps). The audio recording of the semi‐structured interviews on acceptability will be pseudonymised and transferred directly from the recording device to Data Safe Haven. Any participant who opts out of the study will have their data deleted.

An independent data monitoring committee will have access to the unblinded comparative data and will monitor these data. The committee will make recommendations on whether there are any reasons to terminate the trial that the chair will report to the independent trial steering committee.

### Analysis

The data will be analysed using R Studio [53]. The data analyst will be blinded to participants' group and the analysis plan will be finalised and uploaded onto Open Science Framework before the start of data analysis when the trial will be analysed in accordance with the pre‐specified plan.

Descriptive statistics of participants' sociodemographic characteristics and AUDIT score will be reported for whom the study recruited and who then accessed the intervention. The difference between the intervention and comparator groups on baseline characteristics will be assessed using one‐way ANOVAs for continuous variables (age, AUDIT score) and 2‐sided χ^2^ tests (or Fisher's exact test for rare events) for categorical variables (sex, ethnicity, education, occupation, income and COVID‐19 survey measures).

ANOVAs are generally considered robust against small deviations from the normality assumption with only a small effect on the type I error rate [54]. However, if there is evidence of significant deviation we will attempt to resolve this with transformations (e.g. logarithmic or square‐root transformations) or choose the nonparametric Kruskal‐Wallis H test, which does not require the assumption of normality.

#### Analysis of primary and secondary outcomes

The primary analysis will use a conservative intention‐to‐treat approach to missing data with the assumption of no change for participants who do not respond to follow‐up (i.e. analysis of outcome data from all randomised participants). The effect of group allocation on the primary outcome, change in weekly alcohol consumption, will be examined with an adjusted regression model, adjusting for baseline consumption [55, 56, 57].

A secondary analysis will be conducted to assess differences in change in weekly alcohol consumption using an unadjusted regression model. Regression models have several statistical assumptions that will be assessed (i.e. the errors of the data are normally distributed and there is equal variances between treatments). The analyses are robust to slight deviations in parametric assumptions, but where large deviations are found data transformations and appropriate non‐parametric tests will be considered.

Additional sensitivity analyses will be conducted for the primary outcome at 6 months: (i) responders‐only (i.e. those who completed the 6‐month follow‐up survey); (ii) using multiple imputation for non‐responders on baseline characteristics (with five imputed data sets [58] combined using Rubin's rules [59]) and assuming a normal distribution with a mean of 0 and SD reflecting the variation in change among responders; (iii) per‐protocol approach whereby only participants who downloaded Drink Less in the intervention group and those who viewed the NHS alcohol advice webpage in the comparator group are included in the analyses, and whereby participants whose treatment was contaminated are excluded; (iv) an instrument variable analysis accounting for non‐use in the intervention group and contamination in the comparator by operationalising the difference in app usage between the two conditions; (v) last observation carried forward; and (vi) assessing the effect of the condition by recruitment source.

Secondary analyses will assess: (i) the secondary outcomes at 6 months using ANOVA and χ^2^ analyses as appropriate and (ii) the change over time in the primary outcome using 1‐ and 3‐month follow‐up data.

Confidence intervals, effect sizes (partial η^2^ for ANOVA analyses, odds ratios for χ^2^ and regression analyses) and exact *P* values will be reported. Bayes factors will be calculated using a half normal distribution to specify the predicted effect (of a 2 UK unit reduction per week) with a peak at 0 (no effect) and the SD equal to the expected effect size with Robustness Regions reported to specify the range of expected effect sizes that support the same conclusion [60].

Finally, interactions will be assessed between group allocation with age, sex, ethnicity, education, occupation, income and COVID‐19 measures (survey and national responses) for primary and secondary outcomes. Where significant interactions are found the findings will be stratified, drawing on the PROGRESS‐Plus framework to explicitly consider health equity between the intervention and comparator group [61].

#### Process evaluation

The process evaluation will involve quantitative analysis of the psychological and engagement measures and qualitative analysis of interview transcripts relating to the acceptability of the intervention.

The extent of user engagement with Drink Less will be evaluated through descriptive statistics of the engagement measures. Detailed modelling of variations between participants will be conducted to explore variation in engagement and psychological measures by sociodemographic characteristics, baseline AUDIT scores and COVID‐19 measures (survey and national responses). A mediation analysis will be conducted to determine if any effect of group allocation on the primary outcome is mediated by changes in the psychological measures. The psychological measures will also be integrated into the modelling of effectiveness outcomes for Drink Less to identify links between the outcomes, participant engagement and psychological measures.

Anonymised interview transcripts on the intervention's acceptability will be analysed using a combined framework and thematic analysis approach. This involves initially coding participant responses according to the Theoretical Framework of Acceptability construct they are judged to represent best, then grouping similar responses within each construct inductively to generate content themes representing how that construct contributes to reported acceptability. Twenty‐six participants will be interviewed initially (13 from each group) [24], then data will be analysed and data collection will continue in an iterative process (adding 10 participants at a time) until thematic ‘meaning’ saturation is reached. Meaning saturation is defined as the point at which the issues are fully understood and no further dimensions, nuances or insights are found [62].

#### Health economic evaluation

The economic evaluation will take a two‐stage approach to analyse the cost‐utility of Drink Less from the NHS perspective. The first stage will be an analysis of the cost‐effectiveness of the app in the trial population over the duration of the trial itself (including follow‐up). Costs will include the cost of the interventions in both arms and the cost of NHS resource use (i.e. cost of changes in service use and treatments). The cost‐effectiveness analysis will take into account the total development cost of Drink Less but keep it separate from the incremental evaluation because there are no anticipated additional costs per user using the app. Effects will be measured in terms of (i) reduction in alcohol consumption and (ii) health‐related quality of life, measured in quality‐adjusted life years (QALYs). The cost‐utility will be measured in terms of Incremental Cost‐Effectiveness Ratio, the ratio between the difference in costs and difference in effects between the intervention and comparator groups.

As there can be a delay of several years between reductions in alcohol consumption and improvements in health [63], the full impacts of interventions designed to reduce alcohol consumption on health and healthcare costs may not be seen until well beyond the time horizon of an RCT. The second stage of the economic evaluation will address this limitation by using the established and widely‐used Sheffield Alcohol Policy Model [64, 65] to assess the longer‐term cost‐effectiveness of the intervention, if rolled out on a national level through active promotion to the public, over a 20‐year time horizon.

Both short‐ and long‐term evaluations will assess the impact on health inequalities using a Distribution Cost‐Effectiveness Analysis framework [66, 67]. Costs and QALY outcomes will be estimated separately by socioeconomic group, defined by social grade (AB, C1, C2, D or E). These group‐specific results will be combined with estimates of health pre‐intervention and the opportunity cost of additional healthcare spending to place the intervention on the ‘health equity impact plane’. Published estimates of inequality aversion, quantifying the extent to which society is willing to trade off changes in cost‐effectiveness for changes in health inequalities, will be used to identify the optimal strategy after accounting for the inequality impacts of each approach.

### Ethical approval

Ethical approval has been obtained from UCL Research Ethics Committee (16799/001).

### Dissemination policy

Results will be disseminated by open‐access peer‐reviewed journal articles, presentations at scientific conferences, press releases, a stakeholder workshop and blog posts. NIHR authorship guidelines will be followed. Study materials, anonymised data and code will be made available on Open Science Framework (on the project page: https://osf.io/q8mua), and the source code for the app will be released under the GNU General Public License (v3) on GitHub.

## Discussion

This study will provide evidence on the effectiveness and cost‐effectiveness of the Drink Less app—a theory‐ and evidence‐informed intervention—for the reduction of hazardous and harmful alcohol consumption. The embedded mixed‐methods process evaluation will evaluate the extent of user engagement with Drink Less and whether user engagement moderates or changes in psychological measures mediate the effectiveness of the intervention and participants’ views on the acceptability of the intervention. Furthermore, the use of behavioural science in the development and refinement of the Drink Less app and the trial methodology will improve the understanding of what works for whom and why. Drink Less collects detailed user engagement and behavioural data of potential value in advancing its underpinning theory [68], which can inform future behaviour change interventions. The principles of Open Science will continue to be followed, which is important for efficient scientific progress.

Drink Less is in a good position for future implementation in healthcare settings because it is already aligned with the criteria from the NHS Digital Assessment Questionnaire and with the NICE Evidence for Effectiveness Framework and Code of Conduct for Digital Health [69, 70, 71]. The app will be submitted to the NHS Apps Library if found to be effective, which may result in healthcare practitioners recommending or prescribing the app to patients [72]. Drink Less is a standalone app and therefore, in a strong position to be actively promoted on a national level, through media contacts and relevant organisations, to encourage use of the app amongst wider society. A sustainability model will be developed as part of this research project to maximise the impact of the app by enabling long‐term planning for its implementation, adoption and future development and will inform the long‐term economic analysis in terms of potential reach and costs. A current limitation is that Drink Less is only available on iOS devices though this RCT will provide a proof of concept and strong rationale, if effective, for developing a native Android version, which would maximise the potential impact of Drink Less on public health. A potential limitation of the trial is the choice of the NHS website as the comparator instead of an app‐based intervention that would allow us to directly compare the recommendation to download one of two apps. However, using the NHS website as the comparator condition is of direct policy relevance and strengthens the public health impact of the study.

There are two main anticipated challenges with this trial: recruitment and retention rates that we have attempted to minimise as follows. A multi‐pronged recruitment strategy is planned, including placing an advert on a NHS webpage, which provides the ability to access a large number of drinkers. A higher rate of recruitment than is currently required (265 participants per month) was achieved in the factorial trial of Drink Less (355 per month) [13] with web‐based recruitment and screening. However, a contingency plan is in place that involves advertisements placed on Google and Facebook (following the methods used in a similar trial [73]) and use of a recruitment company. We will also calculate Bayes factors, which in the situation of under recruitment will allow us to distinguish between two interpretations of a non‐significant result: (i) support for the null hypothesis of ‘no effect’ and (ii) data are insensitive to detect an effect [74, 75]. To maximise the overall retention rate, financial incentives will be offered as a reasonable acknowledgement of participants' time, and a range of methods will be used for follow‐up contact sequentially, which has been shown to improve response rates [76]. The trial cost per participant and methods of follow‐up contact are comparable with similar studies that achieved high retention rates [73, 77]. The intention‐to‐treat approach to analysis is conservative, meaning that if a follow‐up rate similar to that in other trials is achieved, then if anything the effect size will be an underestimate.

We acknowledge that the research team is evaluating an app that it has developed and that there may be a perceived bias. The team have no financial stake in the app and the code is available in Github by the GNU General Public License (v3). The trial has been designed to meet the Cochrane Risk of Bias criterion of ‘low risk’ and we have followed best practice guidelines to ensure a robust evaluation is conducted, including blinding and oversight by independent trial steering and data monitoring committees. The data will be analysed in accordance with a pre‐specified analysis plan approved by the committees and uploaded onto Open Science Framework before the start of data analysis.

## Conclusions

This study will be the first RCT of an alcohol reduction app for the general population in the United Kingdom, thereby starting to build a strong evidence base on the effectiveness and cost‐effectiveness of health‐related apps. The RCT will be able to directly inform the pragmatic question of interest: whether it is worth investing resources into promoting the Drink Less app.

## Clinical trial registration

ISRCTN64052601.

## Author contributions

**Claire Garnett:** Conceptualization‐Equal, Funding acquisition‐Equal, Methodology‐Equal, Writing‐original draft‐Lead. **Melissa Oldham:** Methodology‐Equal, Writing‐review & editing‐Equal. **Colin Angus:** Conceptualization‐Supporting, Funding acquisition‐Supporting, Methodology‐Equal, Writing‐review & editing‐Equal. **Emma Beard:** Conceptualization‐Supporting, Funding acquisition‐Supporting, Methodology‐Equal, Writing‐review & editing‐Equal. **Robyn Burton:** Conceptualization‐Equal, Funding acquisition‐Supporting, Methodology‐Equal, Writing‐review & editing‐Equal. **Matt Field:** Conceptualization‐Equal, Funding acquisition‐Supporting, Methodology‐Equal, Writing‐review & editing‐Equal. **Felix Greaves:** Conceptualization‐Equal, Funding acquisition‐Supporting, Methodology‐Equal, Writing‐review & editing‐Equal. **Matthew Hickman:** Conceptualization‐Equal, Funding acquisition‐Supporting, Methodology‐Equal, Writing‐review & editing‐Equal. **Eileen Kaner:** Conceptualization‐Equal, Funding acquisition‐Supporting, Methodology‐Equal, Writing‐review & editing‐Equal. **Gemma Loebenberg:** Project administration‐Lead, Writing‐review & editing‐Equal. **Susan Michie:** Conceptualization‐Equal, Funding acquisition‐Supporting, Methodology‐Equal, Writing‐review & editing‐Equal. **Marcus Munafo:** Conceptualization‐Equal, Funding acquisition‐Supporting, Methodology‐Equal, Writing‐review & editing‐Equal. **Elena Pizzo:** Conceptualization‐Supporting, Funding acquisition‐Supporting, Methodology‐Equal, Writing‐review & editing‐Equal. **Jamie Brown:** Conceptualization‐Equal, Funding acquisition‐Equal, Methodology‐Equal, Writing‐review & editing‐Equal.

## Ethics committee approval

16799/001.

## Declarations of interest

C.G., E.P., M.M., M.F., M.O., G.L. and S.M. have no conflicts of interest in undertaking this research. J.B. and E.B. have received unrestricted funding related to smoking cessation research. J.B. sits on the scientific advisory board for the SmokeFree app. M.H. has received unrestricted speaker fees in the last 5 years from MSD, Gillead, Abbvie unrelated to this project. E.K. led two Cochrane Collaboration reviews in the field of screening and brief alcohol interventions including digital interventions and is currently leading an NIHR School of Public Health Research project that involves a network meta‐analysis bringing together both bodies of evidence. M.F. received funding from Alcohol Change UK in 2019 to conduct a rapid evidence review of digital interventions for the reduction of alcohol‐related harm. F.G. is employed by both PHE and Imperial. R.B. is a visiting researcher at King's College London and the Universityv of Southampton and has done consultancy for WHO Europe. C.A. has received funding for commissioned research from Systembolaget, the Swedish government‐owned alcohol retail monopoly and Alko (the Finnish equivalent).

## References

[1] World Health Organisation . Global status report on alcohol and health 2018. 2018.

[2] Brown J., West R., Angus C., Beard E., Brennan A., Drummond C. *et al*. Comparison of brief interventions in primary care on smoking and excessive alcohol consumption: a population survey in England. Br J Gen Pract 2016; 66: e1–e9. 26719481 10.3399/bjgp16X683149 PMC4684029

[3] Nilsen P., Kaner E., Babor T. F. Brief intervention, three decades on. Nord Stud Alcohol Drugs 2008; 25: 453–467.

[4] Wilson G. B., Lock C. A., Heather N., Cassidy P., Christie M. M., Kaner E. F. S. Intervention against excessive alcohol consumption in primary health care: a survey of GPs' attitudes and practices in England 10 years on. Alcohol Alcohol 2011 Sep 1; 46: 570–577. 21690169 10.1093/alcalc/agr067 PMC3156887

[5] Kaner E. F. S., Beyer F. R., Garnett C., Crane D., Brown J., Muirhead C. *et al*. Personalised digital interventions for reducing hazardous and harmful alcohol consumption in community‐dwelling populations. Cochrane Database Syst Rev 2017. 10.1002/14651858.CD011479.pub2 PMC6483779 28944453

[6] West R., Michie S. A Guide to Development and Evaluation of Digital Interventions in Healthcare. London: Silverback Publishing; 2016.

[7] Ofcom Communications Market Report 2018. 2018.

[8] Gajecki M., Berman A. H., Sinadinovic K., Rosendahl I., Andersson C. Mobile phone brief intervention applications for risky alcohol use among university students: a randomized controlled study. Addict Sci Clin Pract 2014; 9: 11. 24985342 10.1186/1940-0640-9-11 PMC4091647

[9] Gajecki M., Andersson C., Rosendahl I., Sinadinovic K., Fredriksson M., Berman A. H. Skills training via smartphone app for university students with excessive alcohol consumption: a randomized controlled trial. Int J Behav Med 2017; 24: 778–788. 28224445 10.1007/s12529-016-9629-9 PMC5608866

[10] Bertholet N., Godinho A., Cunningham J. A. Smartphone application for unhealthy alcohol use: pilot randomized controlled trial in the general population. Drug Alcohol Depend 2019; 195: 101–105. 30611977 10.1016/j.drugalcdep.2018.12.002

[11] Hides L., Quinn C., Cockshaw W., Stoyanov S., Zelenko O., Johnson D. *et al*. Efficacy and outcomes of a mobile app targeting alcohol use in young people. Addict Behav 2018 Feb 1; 77: 89–95. 28992580 10.1016/j.addbeh.2017.09.020

[12] Garnett C., Crane D., West R., Brown J., Michie S. The development of drink less: an alcohol reduction smartphone app for individuals who drink excessively. Transl Behav Med 2018; 9: 296–307. 10.1093/tbm/iby043 PMC6417151 29733406

[13] Crane D., Garnett C., Michie S., West R., Brown J. A smartphone app to reduce excessive alcohol consumption: identifying the effectiveness of intervention components in a factorial randomised control trial. Sci Rep 2018; 8: 4384. 29531280 10.1038/s41598-018-22420-8 PMC5847600

[14] Crane D., Garnett C., Brown J., West R., Michie S. Behavior change techniques in popular alcohol reduction apps: content analysis. J Med Internet Res 2015; 17: e118. 25977135 10.2196/jmir.4060 PMC4468601

[15] Craig P., Dieppe P., Macintyre S., Michie S., Nazareth I., Petticrew M. Developing and evaluating complex interventions: the new Medical Research Council guidance. BMJ 2008; 337: a1655. 18824488 10.1136/bmj.a1655 PMC2769032

[16] Collins L. M., Murphy S. A., Strecher V. The multiphase optimization strategy (MOST) and the sequential multiple assignment randomized trial (SMART): new methods for more potent eHealth interventions. Am J Prev Med 2007; 32: S112–S118. 17466815 10.1016/j.amepre.2007.01.022 PMC2062525

[17] Michie S., Atkins L., West R. The Behaviour Change Wheel ‐ A Guide To Designing Interventions, 1st edn. London: Silverback Publishing; 2014.

[18] Garnett C., Crane D., West R., Brown J., Michie S. Identification of behavior change techniques and engagement strategies to design a smartphone app to reduce alcohol consumption using a formal consensus method. JMIR Mhealth Uhealth 2015; 3: e73. 26123578 10.2196/mhealth.3895 PMC4526967

[19] Michie S., Whittington C., Hamoudi Z., Zarnani F., Tober G., West R. Identification of behaviour change techniques to reduce excessive alcohol consumption. Addiction 2012; 107: 1431–1440. 22340523 10.1111/j.1360-0443.2012.03845.x

[20] Crane D., Garnett C., Brown J., West R., Michie S. Factors influencing usability of a smartphone app to reduce excessive alcohol consumption: think aloud and interview studies. Front Public Health 2017; 5: 1–19. 28421175 10.3389/fpubh.2017.00039 PMC5376568

[21] Brown J., Michie S., Geraghty A. W. A., Yardley L., Gardner B., Shahab L. *et al*. Internet‐based intervention for smoking cessation (StopAdvisor) in people with low and high socioeconomic status: a randomised controlled trial. Lancet Respir Med 2014; 2: 997–1006. 25262458 10.1016/S2213-2600(14)70195-X

[22] Faul F., Erdfelder E., Lang A.‐G., Buchner A. G*power 3: a flexible statistical power analysis program for the social, behavioral, and biomedical sciences. Behav Res Methods 2007; 39: 175–191. 17695343 10.3758/bf03193146

[23] Kaner E. F., Beyer F. R., Muirhead C., Campbell F., Pienaar E. D., Bertholet N. *et al*. Effectiveness of brief alcohol interventions in primary care populations. Cochrane Database Syst Rev 2018. 10.1002/14651858.CD004148.pub4 PMC6491186 29476653

[24] Francis J. J., Johnston M., Robertson C., Glidewell L., Entwistle V., Eccles M. P. *et al*. What is an adequate sample size? Operationalising data saturation for theory‐based interview studies. Psychol Health 2010; 25: 1229–1245. 20204937 10.1080/08870440903194015

[25] Drink Less on iTunes store [Internet]. [cited 2016 Oct 5]. Available from: https://itunes.apple.com/gb/app/drink‐less‐get‐help‐reducing/id1020579244?mt=8

[26] Department of Health . UK Chief Medical Officers' Low Risk Drinking Guidelines. 2016.

[27] NHS: Alcohol support ‐ tips on cutting down [Internet]. [cited 2019 Oct 9]. Available from: https://www.nhs.uk/live‐well/alcohol‐support/tips‐on‐cutting‐down‐alcohol/

[28] Freedland K. E., King A. C., Ambrosius W. T., Mayo‐Wilson E., Mohr D. C., Czajkowski S. M. *et al*. The selection of comparators for randomized controlled trials of health‐related behavioral interventions: recommendations of an NIH expert panel. J Clin Epidemiol 2019; 110: 74–81. 30826377 10.1016/j.jclinepi.2019.02.011 PMC6543841

[29] Department for Work and Pensions . Households Below Average Income: An analysis of the UK income distribution: 1994/95–2014/15. 2019.

[30] CGA . Britain's drinking habits are changing under lockdown [Internet]. 2020 [cited 2020 May 15]. Available from: https://www.cga.co.uk/2020/04/03/britains‐drinking‐habits‐are‐changing‐under‐lockdown/

[31] Alcohol Change UK. Drinking during lockdown. 2020.

[32] Institute of Alcohol Studies . Alcohol consumption during the COVID‐19 lockdown. 2020.

[33] Jackson S. E., Garnett C., Shahab L., Oldham M., Brown J. Association of the Covid‐19 lockdown with smoking, drinking, and attempts to quit in England: an analysis of 2019‐2020 data. *Addiction* 2020. 10.1111/add.15295 PMC8436745 33089562

[34] Coulton S., Perryman K., Bland M., Cassidy P., Crawford M., Deluca P. *et al*. Screening and brief interventions for hazardous alcohol use in accident and emergency departments: a randomised controlled trial protocol. BMC Health Serv Res 2009; 9: 114. 19575791 10.1186/1472-6963-9-114 PMC2712466

[35] Bradley K. A., DeBenedetti A. F., Volk R. J., Williams E. C., Frank D., Kivlahan D. R. AUDIT‐C as a brief screen for alcohol misuse in primary care. Alcohol Clin Exp Res 2007; 31: 1208–1217. 17451397 10.1111/j.1530-0277.2007.00403.x

[36] Bradley K. A., McDonell M. B., Bush K., Kivlahan D. R., Diehr P., Fihn S. D. The AUDIT alcohol consumption questions: reliability, validity, and responsiveness to change in older male primary care patients. Alcohol Clin Exp Res 1998; 22: 1842–1849. 9835306 10.1111/j.1530-0277.1998.tb03991.x

[37] Coulton S., Bland M., Crosby H., Dale V., Drummond C., Godfrey C. *et al*. Effectiveness and cost‐effectiveness of opportunistic screening and stepped‐care interventions for older alcohol users in primary care. Alcohol Alcohol 2017; 52: 655–664. 29016980 10.1093/alcalc/agx065

[38] Coulton S., Perryman K., Bland M., Cassidy P., Crawford M., Deluca P. *et al*. Screening and brief interventions for hazardous alcohol use in accident and emergency departments: a randomised controlled trial protocol. BMC Health Serv Res 2009; 9: 1–10. 19575791 10.1186/1472-6963-9-114 PMC2712466

[39] Kaner E., Bland M., Cassidy P., Coulton S., Deluca P., Drummond C. *et al*. Screening and brief interventions for hazardous and harmful alcohol use in probation services: a cluster randomised controlled trial protocol. BMC Public Health 2009; 9: 1–13. 19922618 10.1186/1471-2458-9-418 PMC2784463

[40] Drummond C., Deluca P., Coulton S., Bland M., Cassidy P., Crawford M. *et al*. The effectiveness of alcohol screening and brief intervention in emergency departments: a multicentre pragmatic cluster randomized controlled trial. PLoS ONE 2014; 9: e99463. 24963731 10.1371/journal.pone.0099463 PMC4070907

[41] Grant K. A., Tonigan J. S., Miller W. R. Comparison of three alcohol consumption measures: a concurrent validity study. J Stud Alcohol 1995; 56: 168–172. 7760562 10.15288/jsa.1995.56.168

[42] Eysenbach G. The law of attrition. J Med Internet Res 2005; 7: e11. 15829473 10.2196/jmir.7.1.e11 PMC1550631

[43] Feinn R., Tennen H., Kranzler H. R. Psychometric properties of the short index of problems as a measure of recent alcohol‐related problems. Alcohol Clin Exp Res 2003; 27: 1436–1441. 14506404 10.1097/01.ALC.0000087582.44674.AF

[44] UKATT Research Team Cost effectiveness of treatment for alcohol problems: findings of the randomised UK alcohol treatment trial (UKATT). BMJ 2005; 331: 544. 16150765 10.1136/bmj.331.7516.544 PMC1200587

[45] Coulton S., Newbury‐Birch D., Cassidy P., Dale V., Deluca P., Gilvarry E. *et al*. Screening for alcohol use in criminal justice settings: an exploratory study. Alcohol Alcohol 2012; 47: 423–427. 22553044 10.1093/alcalc/ags048

[46] Miller E. T., Neal D. J., Roberts L. J., Baer J. S., Cressler S. O., Metrik J. *et al*. Test‐retest reliability of alcohol measures: is there a difference between internet‐based assessment and traditional methods? Psychol Addict Behav 2002; 16: 56–63. 11934087

[47] Shorter G. W., Heather N., Bray J. W., Giles E. L., Holloway A., Barbosa C. *et al*. The ‘outcome reporting in brief intervention trials: alcohol’ (ORBITAL) framework: protocol to determine a core outcome set for efficacy and effectiveness trials of alcohol screening and brief intervention. Trials 2017; 18: 611. 29273070 10.1186/s13063-017-2335-3 PMC5741954

[48] Beard E., Brown J., West R., Drummond C., Kaner E., Michie S. Predictive validity, diagnostic accuracy and test‐retest reliability of the strength of urges to drink (SUTD) scale. Int J Environ Res Public Health 2019; 16: 3714. 10.3390/ijerph16193714 PMC6801575 31581626

[49] de Vocht F., Brown J., Beard E., Angus C., Brennan A., Michie S. *et al*. Temporal patterns of alcohol consumption and attempts to reduce alcohol intake in England. BMC Public Health 2016; 16: 917. 27585991 10.1186/s12889-016-3542-7 PMC5009608

[50] Perski O., Blandford A., West R., Michie S. Conceptualising engagement with digital behaviour change interventions: a systematic review using principles from critical interpretive synthesis. Transl Behav Med 2016; 7: 254–267. 10.1007/s13142-016-0453-1 PMC5526809 27966189

[51] Google Analytics. How a web session is defined in Analytics [Internet]. [cited 2017 Dec 6]. Available from: https://support.google.com/analytics/answer/2731565?hl=en

[52] Sekhon M., Cartwright M., Francis J. J. Acceptability of healthcare interventions: an overview of reviews and development of a theoretical framework. BMC Health Serv Res 2017; 17: 88. 28126032 10.1186/s12913-017-2031-8 PMC5267473

[53] RStudio Team . RStudio: Integrated Development Environment for R. Boston, MA; 2019.

[54] Schmider E., Ziegler M., Danay E., Beyer L., Bühner M. Is it really robust?: reinvestigating the robustness of ANOVA against violations of the normal distribution assumption. Methodology 2010; 6: 147–151.

[55] Van Breukelen G. J. P. ANCOVA versus change from baseline: more power in randomized studies, more bias in nonrandomized studies. J Clin Epidemiol 2006; 59: 920–925. 16895814 10.1016/j.jclinepi.2006.02.007

[56] Clifton L., Clifton D. A. The correlation between baseline score and post‐intervention score, and its implications for statistical analysis. Trials 2019 Dec 11; 20: 43. 30635021 10.1186/s13063-018-3108-3 PMC6330413

[57] Fu R., Haley K., Holmer M. P. H. Change Score or Followup Score? An Empirical Evaluation of the Impact of Choice of Mean Difference Estimates. Rockville, MD: Agency for Healthcare Research and Quality; 2015. 25927135

[58] Graham J. W., Olchowski A. E., Gilreath T. D. How many imputations are really needed? Some practical clarifications of multiple imputation theory. Prev Sci 2007; 8: 206–213. 17549635 10.1007/s11121-007-0070-9

[59] Rubin D. Multiple Imputation for Nonresponse in Surveys. New York: John Wiley & Sons; 2004.

[60] Lakens D., McLatchie N., Isager P., Scheel A., Dienes Z. Improving inferences about null effects with Bayes factors and equivalence tests. J Gerontol Ser B 2018; 75: 45–57. 10.1093/geronb/gby065 29878211

[61] O'Neill J., Tabish H., Welch V., Petticrew M., Pottie K., Clarke M. *et al*. Applying an equity lens to interventions: using PROGRESS ensures consideration of socially stratifying factors to illuminate inequities in health. J Clin Epidemiol 2014; 67: 56–64. 24189091 10.1016/j.jclinepi.2013.08.005

[62] Hennink M. M., Kaiser B. N., Marconi V. C. Code saturation versus meaning saturation. Qual Health Res 2017; 27: 591–608. 27670770 10.1177/1049732316665344 PMC9359070

[63] Holmes J., Meier P. S., Booth A., Guo Y., Brennan A. The temporal relationship between per capita alcohol consumption and harm: a systematic review of time lag specifications in aggregate time series analyses. Drug Alcohol Depend 2012; 123: 7–14. 22197480 10.1016/j.drugalcdep.2011.12.005

[64] Brennan A., Meier P., Purshouse R., Rafia R., Meng Y., Hill‐Macmanus D. *et al*. The Sheffield alcohol policy model ‐ a mathematical description. Health Econ 2015 Oct; 24: 1368–1388. 25270223 10.1002/hec.3105

[65] Angus C., Li J., Romero‐Rodriguez E., Anderson P., Parrott S., Brennan A. Cost‐effectiveness of strategies to improve delivery of brief interventions for heavy drinking in primary care: results from the ODHIN trial. Eur J Public Health 2019 Apr 1; 29: 219–225. 30239676 10.1093/eurpub/cky181

[66] Cookson R., Mirelman A. J., Griffin S., Asaria M., Dawkins B., Norheim O. F. *et al*. Using cost‐effectiveness analysis to address health equity concerns. Value Health 2017; 20: 206–212. 28237196 10.1016/j.jval.2016.11.027 PMC5340318

[67] Asaria M., Griffin S., Cookson R. Distributional cost‐effectiveness analysis: a tutorial. Med Decis Making 2016; 36: 8–19. 25908564 10.1177/0272989X15583266 PMC4853814

[68] Garnett C., Crane D., Brown J., Kaner E., Beyer F., Muirhead C. *et al*. Reported theory use by digital interventions for hazardous and harmful alcohol consumption, and association with effectiveness: meta‐regression. JMIR 2018; 20: e69. 29490895 10.2196/jmir.8807 PMC5856921

[69] NHS Digital . How we assess health apps and digital tools [Internet]. [cited 2019 Nov 6]. Available from: https://digital.nhs.uk/services/nhs-apps-library/guidance-for-health-app-developers-commissioners-and‐assessors/how‐we‐assess‐health‐apps‐and‐digital‐tools

[70] NICE . Evidence standards framework for digital health technologies. NICE;

[71] Department of Health & Social Care . Code of conduct for data‐driven health and care technology [Internet]. [cited 2019 Nov 6]. Available from: https://www.gov.uk/government/publications/code-of-conduct-for-data-driven-health-and-care-technology/initial-code-of‐conduct‐for‐data‐driven‐health‐and‐care‐technology

[72] Powell A. C., Landman A. B., Bates D. W. In search of a few good apps. JAMA 2014; 311: 1851–1852. 24664278 10.1001/jama.2014.2564

[73] Watson N. L., Mull K. E., Heffner J. L., McClure J. B., Bricker J. B. Participant recruitment and retention in remote eHealth intervention trials: methods and lessons learned from a large randomized controlled trial of two web‐based smoking interventions. J Med Internet Res 2018; 20: e10351. 30143479 10.2196/10351 PMC6128955

[74] Jeffreys H. The Theory of Probability, 3rd edn. Oxford: Oxford University Press; 1961.

[75] Dienes Z. Using Bayes to get the most out of non‐significant results. Front Psychol 2014; 5: 781. 25120503 10.3389/fpsyg.2014.00781 PMC4114196

[76] Dillman D. A., Phelps G., Tortora R., Swift K., Kohrell J., Berck J. *et al*. Response rate and measurement differences in mixed‐mode surveys using mail, telephone, interactive voice response (IVR) and the internet. Soc Sci Res 2009; 38: 1–18.

[77] Cunningham J. A., Wild T. C., Cordingley J., Van Mierlo T., Humphreys K. Twelve‐month follow‐up results from a randomized controlled trial of a brief personalized feedback intervention for problem drinkers. Alcohol Alcohol 2010; 45: 258–262. 20150170 10.1093/alcalc/agq009 PMC2857148

